# Social determinants of hypertension and type-2 diabetes in Kenya: A latent class analysis of a nationally representative sample

**DOI:** 10.1371/journal.pone.0221257

**Published:** 2019-08-19

**Authors:** Rahma S. Mkuu, Tamika D. Gilreath, Caroline Wekullo, Gabriela A. Reyes, Idethia S. Harvey

**Affiliations:** 1 Transdisciplinary Center for Health Equity Research, Department of Health & Kinesiology, Texas A&M University, College Station, Texas, United States of America; 2 Post Graduate Studies, Masinde Muliro University of Science and Technology, Kakamega, Kenya; 3 Colorado School of Public Health, Colorado University, Denver-Anschutz Medical Campus, Denver, Colorado, United States of America; Florida International University Herbert Wertheim College of Medicine, UNITED STATES

## Abstract

**Introduction:**

Cardiovascular disease is among the leading causes of death in Kenya and type II diabetes (T2D) is a growing chronic health concern in the country. However, a gap exists in examining how demographic and social characteristics coalesce to identify individuals at high risk for hypertension and/or T2D in Kenya. The current study examined demographic typologies associated with self-report diagnoses.

**Methods:**

Nationally representative cross-sectional study using 43,898 individuals from the Kenya Demographic and Health Survey 2014. Main Outcome Measures were self-reported Hypertension and Type 2 Diabetes diagnosis. Descriptive analyses were conducted using STATA 14. Latent class analysis (LCA) was conducted using Mplus 7.4.

**Results:**

Approximately 5% reported hypertension and 1% reported T2D. Latent class analysis suggested a 4-class solution. The class with the highest likelihood to report previous diagnosis of hypertension (10.4%), consisted of high proportion of married adult women. The second highest prevalence of previous diagnosis of hypertension (4.4%) consisted of a high proportion of married middle aged men with high probability of being smokers. The results suggest that Kenyan women over 30 years may be at increased risk of hypertension compared to men. Future studies should include additional socio-demographic and behavioral characteristics to better understand gender differences in correlates for hypertension to be used for targeted and tailored health promotion-interventions.

## Introduction

Non-communicable diseases (NCDs) are projected to eclipse infectious diseases in half a decade in low-middle income countries (LMICs) [1]. Currently majority of NCD deaths globally, (80%), originate from LMICs [2]. The increasing burden of NCDs in LMICs is attributed to demographic and epidemiologic transitions [3, 4]. Urban populations in LMICS are more likely to be predisposed to decreased physical activity and increased high calorie diets, in their environment, amplifying NCD risk [5, 6]. Additionally, as life expectancy in LMICs increases, so does the risk for NCDs because aging is a major predictor for NCDs [7].

Two major NCDs, hypertension (i.e, high blood pressure) and type-2 diabetes (T2D) are growing challenges in LMICs [1, 8]. Hypertension is the leading risk factor for cardiovascular disease (CVD) [8], the leading cause of death worldwide [9]. The continent of Africa has the highest hypertension prevalence (46%) [10]. The World Health Organization (WHO) reports an overall prevalence of 28.7% for hypertension in Kenya [11]. Type-2 diabetes (T2D), one of the top four priority NCDs identified by the WHO [12], increases risk for CVD [13]. Globally, T2D has almost quadrupled in the past four decades (i.e., from108 million in 1980 to 422 million in 2014) [12]. Moreover, the prevalence of T2D in LMICs, particularly in sub-Saharan Africa is increasing [12, 14].

Kenya is experiencing demographic transition (increased population growth, life expectancy and urbanization). Kenya’s population has almost doubled since 1990 from a population of 23 million to a current population of 46 million and the life expectancy has grown from 58 years to 62 years during this time-period [15]. Additionally, Kenya’s urban population increased from 16.75% in 1990 to 26.62% in 2015 [16]. In Kenya, CVDs are now among the top 10 causes of death in the country [11], resulting in increased NCD research during the past decade [17–21] with urban Kenyans having higher prevalence and risk factors for NCDs [17, 18]. Previous studies in Kenya demonstrated zero or very low hypertension prevalence rates [22, 23], however, a recent study reported, 49.9% prevalence among slum residents [19]. A recent study in Kenya reports T2D prevalence of 5.3% [17]. Reported factors associated with T2D and hypertension in Kenya include, increased age [17, 21], family history [24], living in urban areas [17, 20], and being overweight or obese [17, 25]. Despite the increased body of literature on hypertension and T2D in Kenya, studies are limited to specific population settings: urban slums [18, 20, 26], rural areas [19, 21], and specific regions such as Nairobi [18, 20, 26].

To the best of the authors’ knowledge, no study assesses risk factors for hypertension and T2D in Kenya. Additionally, latent class analysis has not been utilized in Kenya to identify high-risk NCD groups. Latent class analysis (LCA) is optimal for identifying unobserved at risk populations represented in a heterogeneous sample. Regression methods identify specific risk factors associated with an outcome variable, but LCA allows for subgroups with similar characteristics (latent variables) that increase the chance of having the outcome to be identified [27]. The aim of this study is to utilize the most current 2014 Kenya Demographic and Health Survey (KDHS) to demonstrate risk factors of self-reported hypertension and T2D in Kenya using latent class analysis.

## Materials and methods

This study utilized cross-sectional secondary data from the 2014 Kenya Demographic and Health Survey (KDHS). The KDHS is nationally representative and aims to provide demographic and health indicator information for the country every five years [28]. The survey was implemented by the Kenya National Bureau of Statistics from May 2014 to October 2014 in partnership with several organizations [28]. A two-stage stratified random cluster sampling method drawn from the Fifth National Sample Survey and Evaluation Programme of the Kenya National Bureau of Statistics was used [28]. Detailed sampling methods are published in the 2014 KDHS report [28]. For this study, all 12,819 male and 14,735 female participants 15 years to 49 years were included in the latent class analysis.

### Measures

#### Social determinants

Participant characteristics were acquired and assessed using categorical variables: wealth index (i.e., poorest, poor, middle, richer, and richest), age in 5-year intervals (i.e., 15–19, 20–24, 25–29, 30–34, 35–39, 40–44, 45–49 and greater than 50 years), and marital status (i.e., never in union, married, living with partner, widowed, divorced, and separated). Dichotomous variables included biological sex (Female = 1, Male = 2) and smoking behaviors (Yes = 1, No = 0).

#### Diabetes and hypertension

Participants were asked the following two questions: 1) “Have you ever been told by a doctor or health worker that you have raised blood pressure or hypertension?” and 2) “Have you ever been told by a doctor or a health worker that you have raised blood sugar or diabetes?” Participants who answered “yes” were classified as either diagnosed with hypertension or T2D.

### Analysis

Descriptive analysis were conducted on using STATA 14. Latent class analysis (LCA) was used to explore social determinants of T2D and hypertension among Kenyan adults using Mplus 7.4 using maximum likelihood to determine the model with the best number of latent classes. Latent class analysis allows for a series of models starting with a 1-class model to be examined to determine the model with the optimal number of classes (e.g., 2-class, 3-class). The analysis was conducted using a stepwise approach where an increasing number of classes were specified at each step (e.g., 1-class model analysis, then 2-class model analysis) [28]. To determine the number of classes, a series of models was conducted starting with a 1-class model followed by specifying an increased number of classes (e.g., 2-class, 3-class, etc.). Optimal model selection was based upon recommended indices including low adjusted Bayesian Information Criterion (BIC) relative to other models, significant Lo-Mendell-Rubin Likelihood Ratio Test (LMR-LRT), and entropy (quality of classification) [28]. The best-fitting model with 4 a four class solution was identified based on decreasing indices as well as interpretably. The model also had the lowest adjusted BIC, significant LMR LRT, and acceptable entropy. The model also supported existing literature on the scope of NCDs in Kenya.

## Results

### Demographic characteristics

Majority of participants, 70.70% (53.48% in LCA analysis) were women, and were between 15–19 years, 20.25%. Most were married 55.11% and classified as being in the poorest wealth quantile 22.65%. Eight percent of our sample were smokers with men having the highest rate 16.97%. The overall rate of having a previous diagnosis of hypertension was 5.85% with women having a higher rate 8.29% compared to men 3.04%. Approximately one percent of the sample reported previous diagnosis of T2D with both women and men having similar rates as the overall sample. Demographic results are outlined in Table 1 according to gender.

**Table 1:** Descriptive statistics for demographic results by gender.

Variables	Women	%	Men	%	Total	%	P value
**Age**							
15–19	6,078	19.56	2,811	21.93	8,889	20.25	
20–24	5,405	17.39	1,981	15.45	7,386	16.83	
25–29	5,939	19.11	1,942	15.15	7,881	17.95	P< 0.001
30–34	4,452	14.32	1,701	13.27	6,153	14.02	
35–39	3,868	12.45	1,486	11.59	5,354	12.20	
40–44	2,986	9.61	1,198	9.35	4,184	9.53	
45–49	2,351	7.56	895	6.98	3,246	7.39	
>49	0	0.00	805	6.28	805	1.83	
**Marital Status**							
Never in Union	8,575	27.59	5,400	42.12	13,975	31.84	
Married	17,751	57.12	6,439	50.23	24,190	55.11	
Living with Partner	1,285	4.13	254	1.98	1,539	3.51	
Widowed	1,191	3.83	82	0.64	1,273	2.90	P<0.001
Divorced	721	2.32	192	1.50	913	2.08	
Separated	1,556	5.01	452	3.53	2,008	4.57	
**Wealth Index**							
Poorest	7,262	23.37	2,683	20.93	9,945	22.65	
Poorer	5,970	19.21	2,578	20.11	8,548	19.47	
Middle	5,946	19.13	2,636	20.56	8,582	19.55	P<0.001
Richer	5,958	19.17	2,758	21.51	8,716	19.86	
Richest	5,943	19.12	2,164	16.88	8,107	18.47	
**Smokers**							
No	14,329	99.69	10,640	83.03	25,329	91.94	P<0.001
Yes	46	0.31	2,175	16.97	2,221	8.06	
**Hypertension**							
No	13,513	91.71	12,427	96.96	25,940	94.15	P<0.001
Yes	1,222	8.29	390	3.04	1,612	5.85	
**Diabetes**							
No	14,572	98.90	12,690	99.10	27,262	98.99	P = 0.095
Yes	162	1.10	115	0.90	277	1.01	

Note: Determined by Pearson *χ*^2^ test; p values <0.05 are considered significant

The Pearson Chi- square test showed there is statistically significantly difference between men and women on several variables including age categories, marital status, wealth index, smokers’ verses nonsmokers, and hypertension(p<0.001). However, the chi- square test showed no statistically difference between men and women with and without diabetes, (p = 0.095).

Before conducting the analysis, we ran a checked of missingness and found 67% of the data was complete. The highest percentage missing was 33% on hypertension and 27% on diabetes variables. A test of missing completely at random (MCAR) was conducted to determine whether it was okay to delete the missing data. The chi2 results was statistically significant, indicating that deleting the missing data was not reasonable, (χ2 (27) = 43898, p = 0.001). Since the model’s variables of interest (T2D, and hypertension) were binary categorical variables, each model was estimated using weighted least-square parameter estimates as the algorithm is designed to handle missing data in Mplus 7.4 [29].

### Latent class analysis results

After running successive models, the four-class solution was identified as being the best fit for the data. Specifically, this model had the best/lowest sample size adjusted BIC and the last significant LMR-LRT p-value. Table 2 demonstrates results of the models ran with the 4 class solution being the class before LMR LRT being non-significant (class 5, LMR LRT = p = 0.814), and entropy dropping significantly from 0.913 in class 4 to 0.718 in class 5. The class 4 solution was more interpretable as classes were more distinct compared to class 3 solution, thus the class 4 solution was identified as the model with the best fit. Class 1 is best described as “healthy young adults” and comprised 11% of the sample. Those assigned to class 1 were likely to be never married young adults between the ages of 20 to 29 years old who did not smoke and who had a low likelihood of reporting hypertension or T2D. Class 2 were married men, who were 30 years and older with a 25% chance of being a smoker. Their likelihood of hypertension or T2D was similar to the rates in the overall sample. Class 3 were married adult women ages 30 and up (53%) who were not likely to be smokers. This class 3, however, had the highest likelihood for hypertension diagnosis (10.4%) and T2D diagnosis (1.3%). Class 4 were adolescent/emerging adults who were most likely to be under 20 years old. These youths were overwhelmingly never married and had the lowest risk of hypertension or T2D. Latent class analysis results are outlined in Tables 2 and 3.

**Table 2:** Best fitting models.

Model	Description	Hypertension		
		Adjusted BIC	LMR LRT	Entropy
			p-value	
1	One-class	496213.1	<0.001	–
2	Two-class	471284.4	<0.001	0.929
3	Three-class	464097.5	<0.001	0.952
4	**Four-class**	**463059.9**	**<0.001**	**0.913**
5	Five-class	462467.5	0.814	0.718

Notes: Best fitting models identified in **bold**. BIC–Bayesian Information Criterion. LMR LRT–Lo-Mendell-Rubin Likelihood Ratio Test p-value for (K-1)-classes. A significant p-value indicates that the (K-1)-class model should be rejected in favor of a model with at least K-classes.

**Table 3:** Probability scales results.

	Healthy Young Adults	Married Adult Men	Married Adult Women	Adolescent/Emerging Adults
Class Prevalence	10.90%	17.10%	52.60%	19.40%
**Variables**				
**Wealth Index**				
Poorest	0.095	0.216	0.252	0.229
Poor	0.134	0.200	0.192	0.225
Middle	0.185	0.195	0.186	0.225
Richer	0.264	0.210	0.188	0.185
Richest	0.323	0.178	0.181	0.135
**Age**				
15–19	0.000	0.001	0.037	0.877
20–24	0.591	0.045	0.150	0.123
25–29	0.318	0.165	0.231	0.000
30–34	0.076	0.207	0.185	0.000
35–39	0.015	0.195	0.166	0.000
40–44	0.000	0.160	0.129	0.000
45–49	0.000	0.120	0.102	0.000
>49	0.000	0.108	0.000	0.000
**Marital Status**				
never union	0.895	0.039	0.039	0.993
married	0.045	0.847	0.763	0.003
living partner	0.043	0.023	0.05	0.003
widowed	0.000	0.011	0.051	0.000
divorced	0.000	0.025	0.031	0.000
separated	0.017	0.055	0.065	0.001
**Smoking Status**				
non smokers	0.901	0.754	0.997	0.992
smokers	0.099	0.246	0.003	0.008
**Hypertension**				
No Hypertension	0.977	0.956	0.896	0.988
Hypertensive	0.023	0.044	0.104	0.012
**Diabetes**				
No Diabetes	0.994	0.987	0.987	0.997
Diabetic	0.006	0.013	0.013	0.003
**Sex**				
Male	0.510	1	0	0.353
Female	0.490	0	1	0.647

## Discussion

This study aimed to identify patterns of self-reported hypertension among a nationally representative Kenyan population. The class with the highest likelihood to report previous diagnosis of hypertension (10.4%) comprised mostly of married adult women. Our results align with previous findings that women have a higher prevalence of hypertension or T2D than males [18, 20]. Among rural dwellers, however, the prevalence of hypertension was twice as high in men (11.9%) compared to women (6.3%) [30]. Our reported prevalence of T2D was 1.3%, this low prevalence might be due to low screening rates. In a previous Kenyan study, only 10.5% of adults reported to ever have had a blood sugar measurement [17].

The higher rates of hypertension and T2D among women may be explained by higher overweight or obesity among women compared to men in Kenya [17]. Overweight or obese Kenyan women are at higher risk of hypertension [17, 26] or T2D [30]. The high-risk women were mostly above the age of 30, relatively young compared to studies demonstrating higher risk for hypertension among older participants [17, 26]. Additionally, the women were spread across wealth classes supporting evidence of high prevalence of hypertension among the highest [21] and lowest wealth classes [18, 20]. The dynamic relationship between wealth and chronic disease may be explained by the double burden of under nutrition and obesity among women both high and low-income women [31, 32].

Smoking, a leading risk factor for morbidity and mortality worldwide [33] has also been shown to influence hypertension rates in Kenya [18]. Our findings are similar to Kenyan studies showing low rates of smoking among women [20, 26]. Despite not engaging in smoking, the women still faced high rates of hypertension or T2D. More studies are therefore needed to explore risk factors among women. Similar to married women, the married men were spread among the wealth index classes showing that hypertension might indeed not just be a problem of those in the highest wealth index. Smoking, as in previous studies [17, 20] was high among the men calling for interventions to curb smoking rates among men.

Overall, the class with the highest proportion of hypertension consisted of a high proportion of married women, between the ages of 25 and 34 years of age, having individuals who are in the middle class or higher wealth status, and non-smokers. In comparison, the classes with the lowest proportion of hypertension, class 1 and class 4 consisted mostly of young people. Class 1 consisted mostly of young people between the ages of 20–29 year old’s, non-smokers who were middle class or higher. Being young in addition to not smoking may influence low proportion of hypertension in the group. Class 4, also contain low proportion of hypertension, mostly consisted of young people between the ages of 15–19 years of age, never married and individuals who were in the poorest or poor wealth categories. The latent classes were generally clustered around age, smoking, and wealth index calling for more studies to understanding how the factors drive differences in hypertension outcomes.

Our study has some limitations. First, the cross-sectional nature of the survey limits understanding of time trends. Both hypertension and T2D were self-reported; not all individuals may have had access to screening and responses might have been affected by recall bias. Due to use of secondary data, we had limited control of variables, we were unable to assess frequency of smoking as well as physical activity. Our preliminary analysis found no significant relationship between physical activity and hypertension and T2D, as found in previous Kenyan studies [18, 20]. Having variables measuring frequency might have allowed for more extensive exploration. Body mass index was only obtained for women but not males thus limiting our analysis of a very important risk factor for hypertension and T2D [34]. Moreover, we did not have access to participant’s nutritional intake. Despite the limitations, our study has some strengths. To the best of our knowledge, this study is the first nationally representative investigation in Kenya on hypertension and T2D, which is the greatest asset of this study. The use of latent class analysis allowed for identification of high-risk groups for hypertension and T2D. The data resulting from demographic health surveys are widely utilized and reputable among scholars and practitioners in the field [35, 36].

Our study has implications for both research, policy and practice. Given the limitations of this study, there is a need for longitudinal studies or datasets focused on NCDs in Kenya to better allow for examination of NCD and risk factors and trends across time. A comprehensive nationally representative dataset which includes objectively measured, 1) risk factors such as high blood pressure or glucose readings, 2) measures and behaviors associated with NCDs such as socioeconomic status, physical activity, nutritional intake, and 3) factors unique to the cultural setting is needed. This study’s attempt to examine how social determinants and NCDs clustered to determine most at-risk groups was a start to building a foundation for understanding the scope of NCD burden in Kenya. More robust studies are needed to understand at risk groups beyond basic social determinants, for example further examining how social positioning, stress, and other factors may influence increase burden of high blood pressure in some groups such as women. Understanding health decision making and access to NCD prevention and care may also provide insight when examining measures of self-reported NCDs. For example, this study relied on self-report of diagnosis of high blood and type 2 diabetes, understanding differences if any in how men and women seek care may have strengthen the conclusions of this study.

This study also has implications for policy and practice. The Kenya National Strategy for the Prevention and Control of Non-Communicable Diseases 2015–2020 aims at “attaining the highest possible standard of health in a manner responsive to the health needs of the population” [37]. The policy provides strategic directions towards reducing the burden of NCDs in Kenya including promoting healthy lifestyles to tackle unhealthy diets, physical inactivity, harmful alcohol use and tobacco use as well as promoting and conducting research and surveillance to prevent NCDs [37]. Results of this study could inform effective strategies that are targeted and tailored to women as they have higher prevalence of overweight and obesity related to increased risk of hypertension.

## Conclusion

In this study, we found that married women over the age of 30 had the highest likelihood of reporting previous T2D or hypertension diagnosis. We have three recommendations: 1) We propose more behavioral questions on factors found to influence chronic disease risk in the demographic health surveys. 2) We suggest efforts to increase screening rates and 3) Finally, gender-specific culturally appropriate health-promotion strategies are needed to curb hypertension and T2D in Kenya.
